# Cancer immunotherapy trial registrations increase exponentially but chronic immunosuppressive glucocorticoid therapy may compromise outcomes

**DOI:** 10.1093/annonc/mdx181

**Published:** 2017-04-14

**Authors:** C. M. Connell, S. Raby, I. Beh, T. R. Flint, E. H. Williams, D. T. Fearon, D. I. Jodrell, T. Janowitz

**Affiliations:** 1 Department of Oncology, University of Cambridge, Addenbrooke’s Hospital, Cambridge; 2 Cancer Research UK Cambridge Institute, University of Cambridge Li Ka Shing Centre, Cambridge, UK; 3 Cold Spring Harbor Laboratory, Cold Spring Harbor, New York; 4 Weill Cornell Medical College, New York, USA

T cell checkpoint-targeted immunotherapy is effective in multiple cancers, but only in subsets of patients [1]. Failure of immunotherapy may be secondary to tumour intrinsic and/or systemic factors that impair immune response. Glucocorticoid administration has known systemic immunosuppressive effects [2] with potential to impair immunotherapy outcome [3], and should therefore be regulated at patient enrolment.

We performed a cross-sectional analysis of T cell checkpoint-targeted cancer immunotherapy trials in solid malignancies registered on the U.S. National Institutes of Health (NIH) trial registry (clinicaltrials.gov) by October 7, 2016. Trials were searched by study type, condition, and interventions targeting the T cell checkpoint proteins CTLA-4, PD-1, PD-L1, PD-L2, LAG3, B7-H3, CD137, OX40, CD27 and GITR. Trials were reviewed manually and independently by two clinicians and registered data on glucocorticoid administration within enrolment criteria recorded.

**Figure 1.**
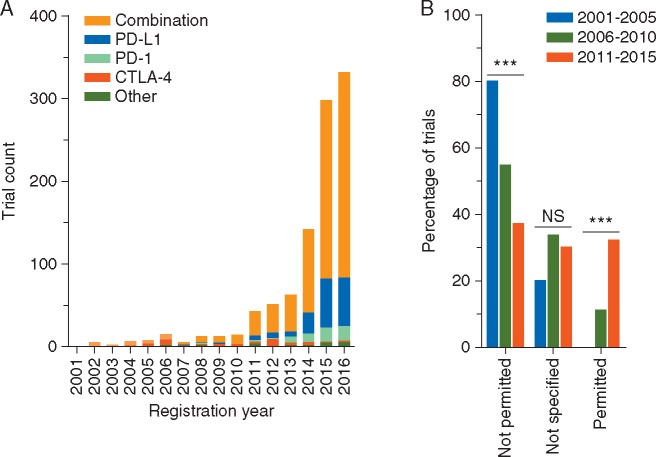
Longitudinal registration count and glucocorticoid administration in T cell checkpoint-targeted cancer immunotherapy trials. (A) Annual registration count of T cell checkpoint-targeted cancer immunotherapy trials. Trials registered by October 7, 2016 on the U.S. National Institutes of Health (NIH) trial registry were categorized according to year of registration and checkpoint protein target. T cell checkpoint proteins targeted in fewer than 10 trials are grouped as ‘other’, and include single agent OX40, GITR, CD137, B7-H3, LAG3, PD-L2, CD27 and trials comparing multiple checkpoint-targeting agents. ‘Combination’ trials include all pre-defined T cell checkpoint-targeted agents used in combination with another agent. (B) Specification of glucocorticoid administration within enrolment criteria of T cell checkpoint-targeted cancer immunotherapy trials. Trials registered on the U.S. NIH trial registry between 2001 and 2015 were categorized according to the specification of chronic systemic glucocorticoid administration within registered patient enrolment criteria. Univariate analysis for data presented was performed using the Cochran–Armitage test for trend assuming monotonical change over time and expected frequencies were met (80% of expected frequencies >5). ***P <* *0.001; NS= not significant.

We identified 1017 registered T cell checkpoint-targeted cancer immunotherapy trials. The number of registrations has progressively increased, exponentially between 2010 and 2015 (*R*^2^ = 0.95) (Figure 1A). For the completed years, 2001–2015, chronic glucocorticoid administration was stated as an exclusion criterion in 40% (276/685), permitted in 29% (201/685) and not specified in 30% (208/685) of trial registration details. The proportion of trials that did not allow glucocorticoid use has decreased significantly (*P *<* *0.001), while the proportion allowing glucocorticoid use has increased significantly (*P *<* *0.001) (Figure 1B). Of the trials permitting glucocorticoid use, the maximum permitted dose of prednisolone equivalent per day was up to 10 mg in 57% of trials (115/201), over 10 mg in 4% of trials (9/201) and not specified in 14% of trials (28/201); 24% of trials (49/201) permitted chronic glucocorticoid use for physiological replacement.

These findings are concerning. The immunosuppressive effects of glucocorticoids are dose dependent, starting at less than 10 mg of prednisolone per day [2], and may be compounded by hypoalbuminaemia present in patients with cancer [4]. Moreover, our pre-clinical work has demonstrated that low dose glucocorticoid administration is sufficient to suppress response to cancer immunotherapy [3]. Therefore, unregulated glucocorticoid administration may result in treatment failure independent of the T cell checkpoint-targeted agent or tumour type.

The use of glucocorticoids as appetite stimulants and anti-emetics, particularly relevant in combination trials with emetogenic chemotherapy or radiotherapy, may also be immunosuppressive and will require critical review. While the use of glucocorticoids for adrenal replacement or chronic immune illness may be unavoidable, stratification for their use at enrolment should be considered.

In addition to glucocorticoid administration, endogenous glucocorticoid levels may also impact on response to immunotherapy. Monitoring and stratification according to baseline glucocorticoid levels, or clinical surrogates of these such as longitudinal weight measurements [3], may yield predictive and prognostic markers of response.

We note that the chronic use of glucocorticoids should be considered independently from the use of glucocorticoids in managing immune-related adverse events during immunotherapy. In fact, the positive correlation of autoimmune side effects and treatment efficacy [5] provides further rationale for considering the role of systemic immunomodulatory variables in determining response to immunotherapy.

Our study is limited by the exclusive use of data from the U.S. NIH trial registry. However, this is the largest clinical trial registry, and the registration of key inclusion and exclusion criteria is international standard [6] and has been mandatory for consideration of publication by the International Committee of Medical Journal Editors member journals since 2005.

In summary, we find glucocorticoid administration to be a neglected immunomodulatory variable in cancer immunotherapy trials, and suggest striving for greater harmony in the monitoring and regulation of systemic glucocorticoids to improve outcomes in cancer immunotherapy.
